# IR and electrochemical synthesis and characterization of thin films of PEDOT grown on platinum single crystal electrodes in [EMMIM]Tf_2_N ionic liquid

**DOI:** 10.3762/bjoc.11.40

**Published:** 2015-03-13

**Authors:** Andrea P Sandoval, Marco F Suárez-Herrera, Juan M Feliu

**Affiliations:** 1 Departamento de Química, Facultad de Ciencias, Universidad Nacional de Colombia, Cra. 30# 45-03, Edificio 451, Bogota, Colombia; 2 Departamento de Química Física e Instituto Universitario de Electroquímica, Universidad de Alicante, Apartado 99, E-03080 Alicante, Spain

**Keywords:** AFM, [EMMIM]Tf_2_N ionic liquid, in situ IR, PEDOT, Pt single crystals

## Abstract

Thin films of PEDOT synthesized on platinum single electrodes in contact with the ionic liquid 1-ethyl-2,3-dimethylimidazolium triflimide ([EMMIM]Tf_2_N) were studied by cyclic voltammetry, chronoamperometry, infrared spectroscopy and atomic force microscopy. It was found that the polymer grows faster on Pt(111) than on Pt(110) or Pt(100) and that the redox reactions associated with the PEDOT p-doping process are much more reversible in [EMMIM]Tf_2_N than in acetonitrile. Finally, the ion exchange and charge carriers’ formation during the p-doping reaction of PEDOT were studied using in situ FTIR spectroscopy.

## Introduction

Conducting polymers have been subject of an intense research during the last decades because they exhibit high conductivity and interesting optical properties. These properties allow their use in several electronic devices [1–2]. The poly(3,4-ethylenedioxythiophene) (PEDOT) is one of the most conducting and stable (thermal and chemical) conducting polymer. For these reasons PEDOT thin films have been extensively studied [3].

Special attention has been paid to the use of electrochemical methods to synthesize conducting polymers due to the high degree of control afforded during the polymerization reaction. On the other hand, thin films of conducting polymers are more suitable to explore electrochemical properties such as stability, capacitance, resistance, ionic exchange kinetics and electrocatalysis [4].

Besides the synthesis technique, there are two important factors that can influence the polymer properties: the surface structure of the electrode and the solvent used [2,5]. It has been reported that the nucleation and growth kinetics, and the electrochemical properties, as the ionic resistance or the electrocatalytic activity, of PEDOT are affected by the surface energy state of the electrode [6]. For example, it was found that the ionic resistance of the PEDOT films electrochemically synthesized on platinum electrodes increases in the order Pt(100) < Pt(110) < Pt(111) [6]. The synthesis of other conducting polymers on well-defined surfaces [7–10] and templates [11] also has shown how the surface affects their adhesion, coverage, morphology and redox kinetics.

On the other hand, the synthesis of conducting polymers in ionic liquids (ILs) has shown an enhancement in stability, organization and electroactivity [12–14]. These characteristics are obtained mainly because of the dry medium, their wide electrochemical window, and their low nucleophilicity. Therefore, during the electrosynthesis of conducting polymers the overoxidation of the polymer is less probable and the average length of the polymer chains is higher in ionic liquids than in molecular solvents [15].

In this work, Pt single crystals electrodes and the moisture stable IL 1-ethyl-2,3-dimethylimidazolium triflimide, [EMMIM]Tf_2_N [16], are used to carry out the electrochemical synthesis of PEDOT. The electrochemical properties of thin films of PEDOT are subsequently studied and compared with the behavior obtained in acetonitrile.

## Results and Discussion

### Cyclic voltammetry

**Figure 1:**
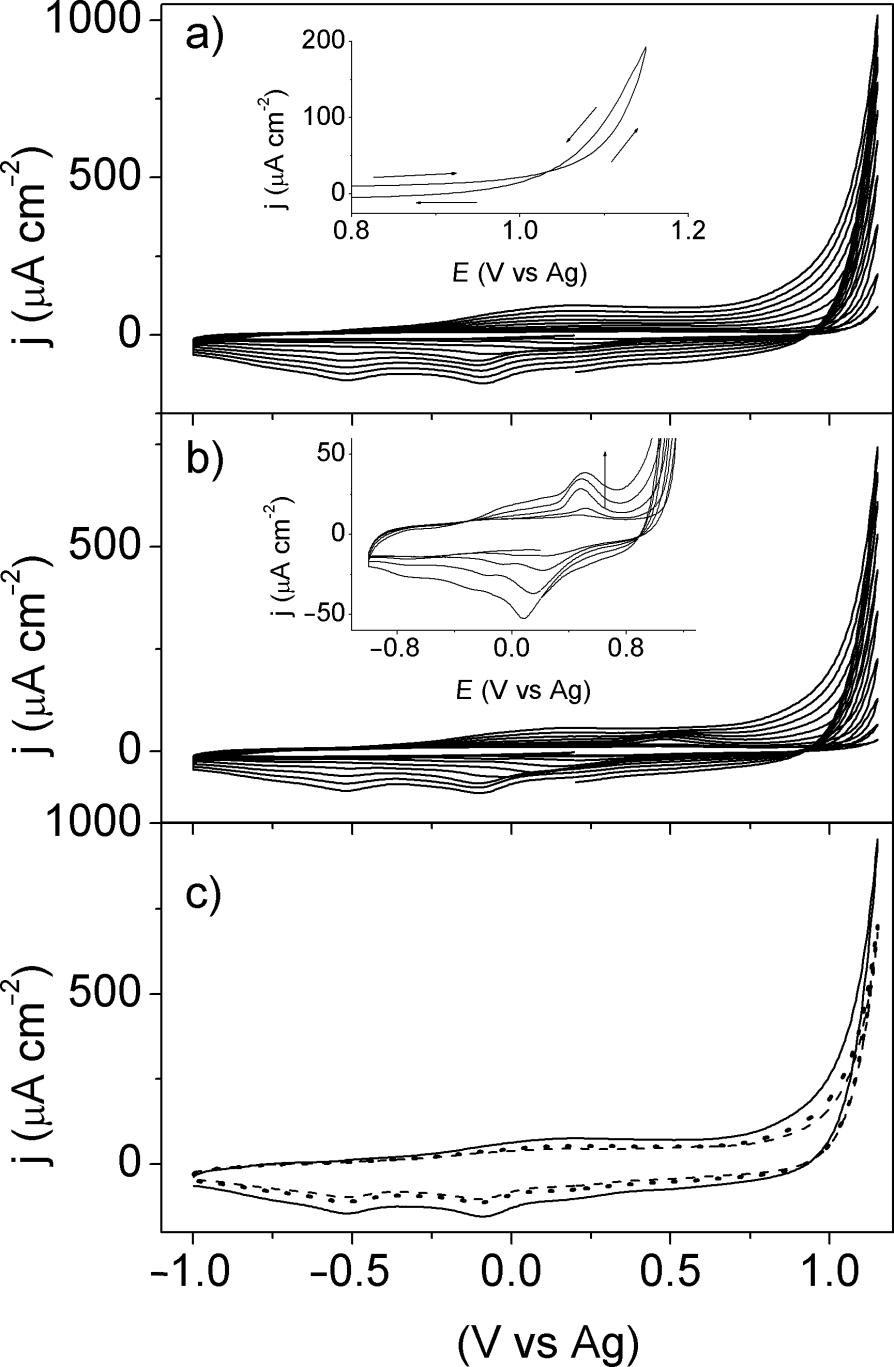
a, b) Cyclic voltammograms of 0.1 M EDOT in [EMMIM]Tf_2_N at 100 mV s^−1^. 10 cycles are shown: a) Pt(111), insert – 2^nd^ cycle; b) Pt(100), Insert – zoom of the cycles from 2 to 6, c) 10^th^ scan of figures “a” and “b” on Pt(111) (solid line), Pt(100) (dashed line) and Pt(110) (dotted line).

Figure 1 shows the cyclic voltammograms of EDOT in [EMMIM]Tf_2_N. The onset of the EDOT oxidation begins at 0.9 V in Pt(111) and Pt(110) while in Pt(100) it starts at 1.0 V. The loop observed during the first scans (Figure 1a – insert) is typical of the electrosynthesis of conducting polymers both in organic media [17] and in ILs [2,18–19]. This characteristic has been well described by Heinze et al. [18]. They associated the loop with an autocatalytic mechanism in which the oligomers formed during the first oxidation cycle react as redox mediators with the monomer, but this kind of loops is also characteristic of a nucleation and growth mechanism. The continuous increase of the current between −1.0 and 1.0 V with the number of cycles clearly indicates that the polymer film is growing. After ten cycles, the currents are higher for the film grown in Pt(111) than in Pt(110) or Pt(100) as it can be observed in Figure 1c. This behavior can be related to the fact that the Pt(111) is more catalytic for the electrodeposition of conducting polymers as it was observed in acetonitrile [6,9].

It is interesting to notice that during the first cycles the voltammograms show one oxidation peak and two negative peaks. In the case of Pt(111) and Pt(110) the oxidation peak appears at 0.37 V and the reduction peaks at 0.31 V and −0.10 V, which rapidly merge to one broad oxidation peak at 0.30 V and two reduction peaks at −0.10 V and −0.50 V. However, in Pt(100) a peak grows at 0.50 V upon potential cycling, while another one appears at 0.30 V, as it can be observed in the Figure 1b (insert graph). On the other hand, the two cathodic peaks at 0.30 V and −0.03 V shift to lower potentials up to the values of −0.10 V and −0.50 V, i.e., the same potentials observed for Pt(111) and Pt(110).

The peaks at 0.50 V and 0.30 V in Pt(100) can be produced by hydrogen oxidation and protons reduction, respectively, because these peaks appear at the same potentials where these reactions take place in [EMMIM]Tf_2_N on Pt(100) [20]. Protons can be produced during the oxidation of EDOT and they can be reduced to hydrogen at negative potentials. These hydrogen reactions can inhibit the polymer growth on Pt(100) during the first cycles. Figure 1 clearly shows that the electrochemical polymerization of EDOT on platinum electrodes is a surface sensitive reaction. It was found that Pt(111) has the highest electrocatalytic activity for the EDOT oxidation reaction followed by Pt(110) and finally Pt(100).

Taking into account that more reproducibility during the synthesis of conducting polymers is normally obtained using the galvanostatic method, the following experiments were done using PEDOT films galvanostatically synthesized.

**Figure 2:**
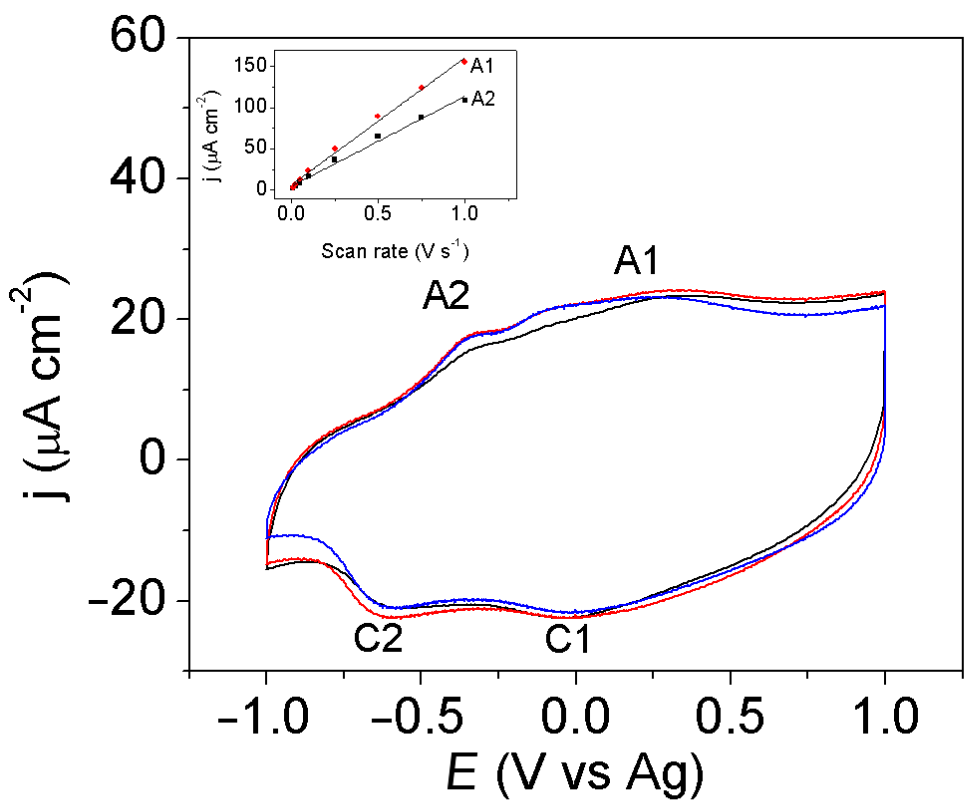
Cyclic voltammograms of PEDOT on Pt(111) (black line), Pt(100) (red line), Pt(110) (blue line) in [EMMIM]Tf_2_N Scan rate 100 mV s^−1^. The films were synthesized applying a charge density of 1.2 mC cm^−2^ during a chronopotentiometry. Insert graph: Current density vs scan rate of PEDOT on Pt(111).

Figure 2 shows the voltammograms of PEDOT in [EMMIM]Tf_2_N free of monomer, in the potential range where the p-doping reaction is observed. Two broad oxidation and reduction signals are present after several cycles, as it was previously reported [19,21–22]. Domagala et al. [23] have provided strong experimental evidences that polarons represent the main charge carrier group in p-doped PEDOT. However, it is still unclear if the two oxidation signals in ionic liquids correspond to two different redox states (polarons and bipolarons) or if they are related to different polymer structures [4].

**Figure 3:**
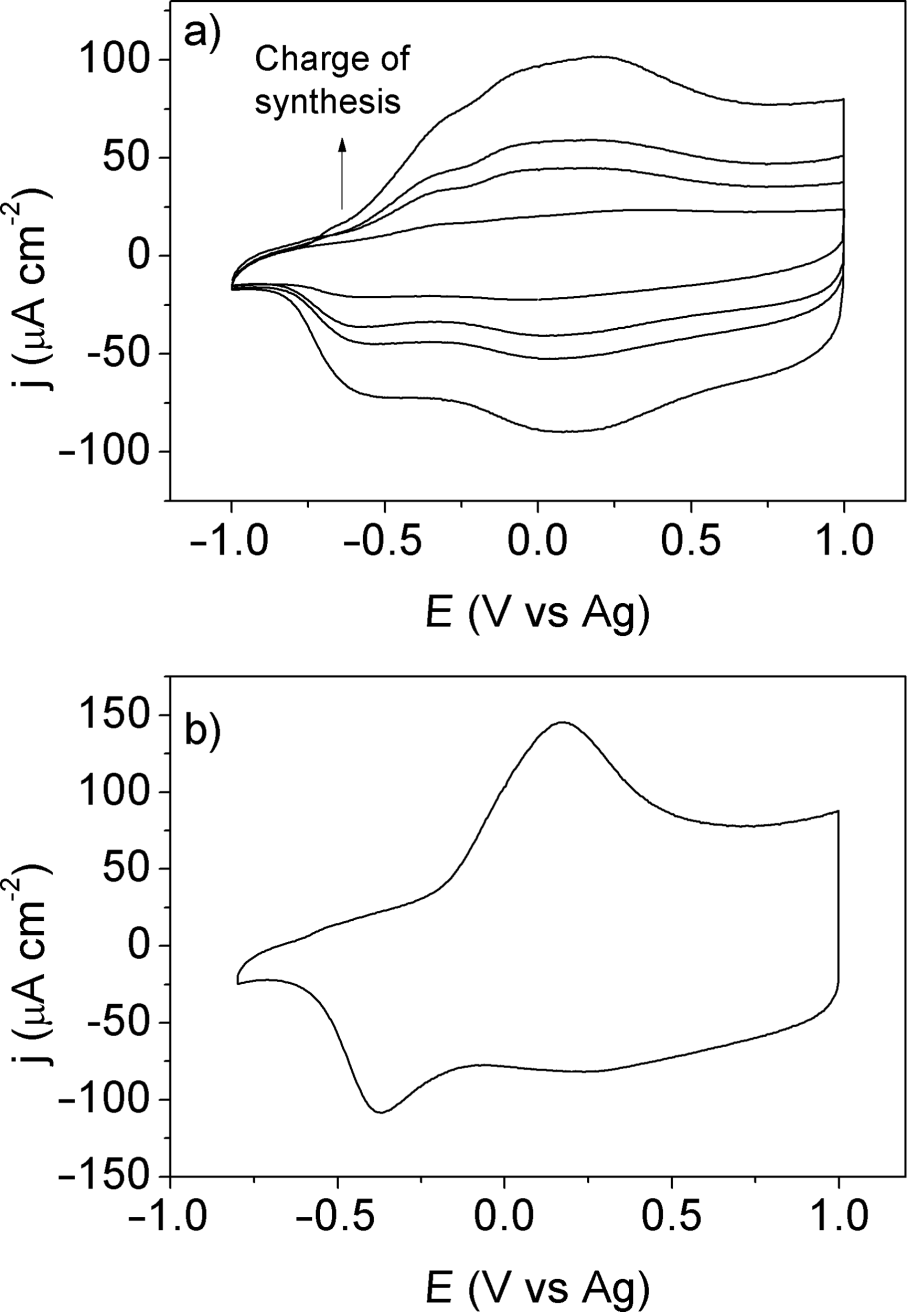
a) Cyclic voltammograms of PEDOT on Pt(111) in [EMMIM]Tf_2_N. The total charge densities used for the galvanostatic synthesis were 1.2, 3.0, 4.3 and 6.2 mC cm^−2^, respectively. The scan rate was 100 mV s^−1^ and the 50^th^ scan is shown. b) Cyclic voltammogram of PEDOT on Pt(111) characterized in 0.1 M [EMMIM]Tf_2_N in acetonitrile. The total charge used during the PEDOT synthesis was 6.2 mC cm^−2^. The scan rate was 100 mV s^−1^.

Voltammetric profiles of PEDOT thin films in Figure 2 show that there are no significant differences between the Pt electrodes. On the other hand, the currents measured at 0.35 and −0.30V (A1 and A2) follow an almost linear tendency with the scan rate, which is characteristic of the surface controlled processes (Figure 2 – insert graph). As expected, thicker PEDOT films were obtained when the synthesis time was increased (Figure 3a).

Figure 3b shows the voltammetry of a PEDOT film synthesized as in Figure 2, but transferred into a cell with 0.1 M [EMMIM]Tf_2_N in acetonitrile as supporting electrolyte. Many differences are observed between Figure 3a and Figure 3b for the PEDOT films of the same thickness: the ratio between height of the cathodic peaks is different, the separation between the anodic and the cathodic peaks is smaller in pure [EMMIM]Tf_2_N and the polymer is oxidized at lower potential in [EMMIM]Tf_2_N than in acetonitrile.

### Chronoamperometry

**Figure 4:**
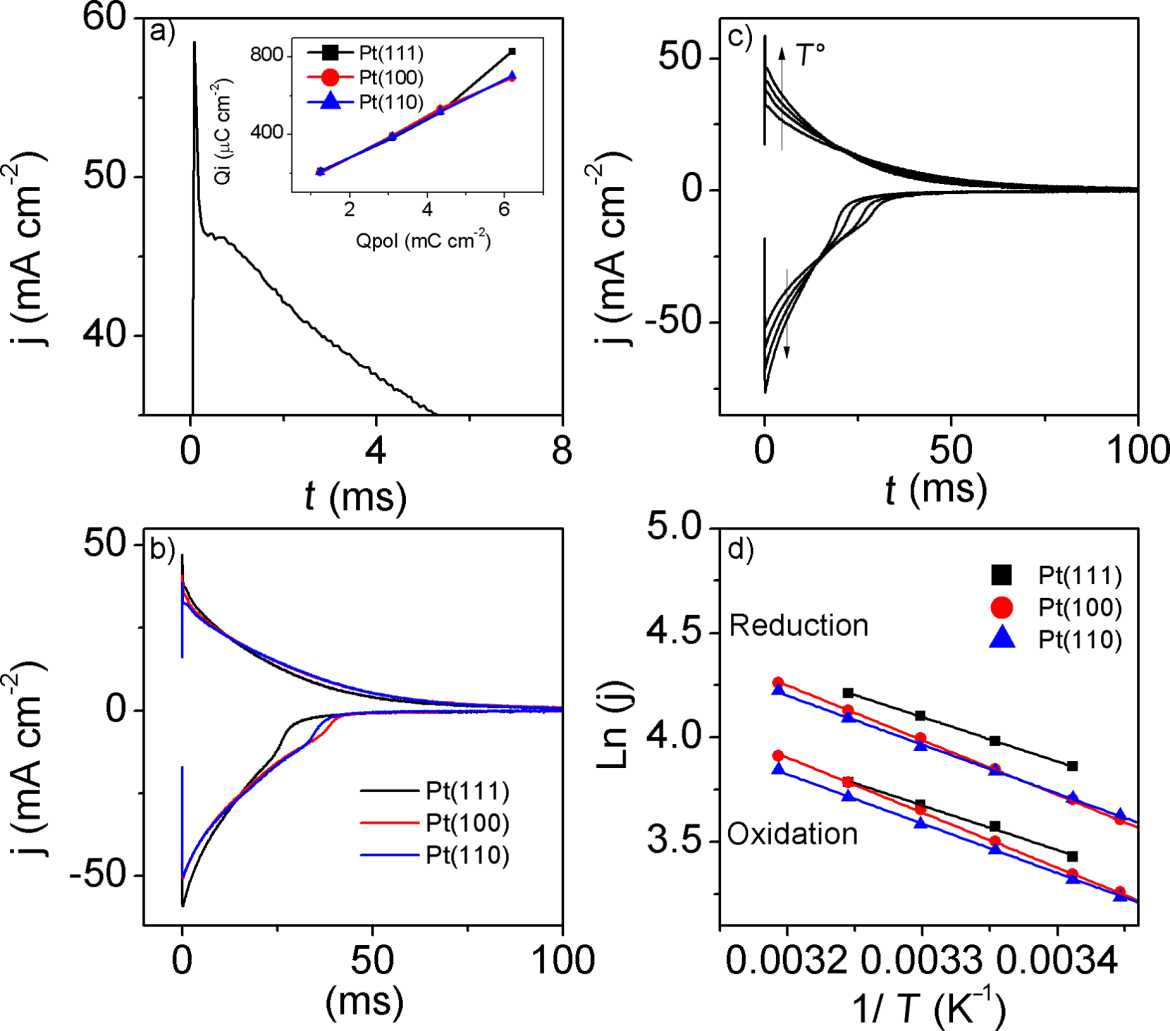
Chronoamperometry of PEDOT films synthesized on platinum single crystals in [EMMIM]Tf_2_N. The total synthesis charge was 6.2 mC cm^−2^. The potential steps were between −1.0 V and 1.0 V. a) Zoom of the first milliseconds for the PEDOT film on Pt(111) at 35 °C. Insert: integrated charge of the chronoamperometry vs the synthesis electric charge for the Pt(hkl) electrodes. b) Chronoamperometry of PEDOT on Pt(hkl) at 25 °C. c) Chronoamperometry of PEDOT on Pt(111) at temperatures of 20, 25, 30, and 35 °C. c) Logarithm of the current at 1.5 ms vs the inverse of temperature for: Pt(111) (black line), Pt(100) (red line) and Pt(110) (blue line).

In order to maintain electroneutrality, the generation of charges in the conducting polymers must be accompanied with the entrance of counterions from the solution. The chronoamperometric experiments allow the study of the ionic exchange kinetics. Figures 4a–c show the current traces when the potential is switched from −1 V to 1 V and vice versa. It is observed that the transients of PEDOT films on the three Pt surfaces used are characteristic of nucleation kinetics [21].

The insert of Figure 4a shows the integrated charge, *Q*_i_, as a function of the charge of synthesis, *Q*_pol_. It can be observed that the charge involved in the current transient increases linearly with increasing polymerization charge. The reduction charge is slightly lower than the oxidation charge. It seems that during the oxidation step the film is slightly overoxidized. No significant differences were observed in the exchange kinetics for the different Pt surfaces (Figure 4b).

Figure 4c shows the current transients at different temperatures for the oxidation and reduction steps. The increase in temperature favors these processes as it has been observed for other conducting polymers [24–25]. With the value of the current at the very beginning of the transient (1.5 ms) it is possible to establish a relation between the current and the temperature using the “initial rate” approximation and the Arrhenius Equation. Since the logarithm of the current density vs the inverse of the temperature plot showed a linear dependence, Figure 4b, the apparent activation energy were calculated (Table 1).

**Table 1:** Nucleation activation energies calculated from the linear graphs shown in Figure 4d.

		Intercept		Slope			Activation energy
	
Electrode		Value	Standard error	Value	Standard error	R^2^	kJ mol^−1^

Pt(111)	Oxidation	10.70	0.35	−2130.27	106.15	0.993	17.71
	Reduction	11.08	0.07	−2116.69	22.11	1.000	17.60

Pt(100)	Oxidation	12.34	0.12	−2636.36	35.68	0.999	21.92
	Reduction	12.64	0.10	−2620.68	29.84	0.999	21.79

Pt(110)	Oxidation	11.39	0.08	−2364.92	22.83	0.999	19.66
	Reduction	11.69	0.10	−2341.47	28.85	0.999	19.47

The values of the activation energy for the oxidation and reduction processes are very similar and very close to the one reported for polythiophene (16 kJ mol^−1^) [24], which means that the oxidation and reduction of PEDOT (and the corresponding counterion exchange) occurs easily in spite of the fact that the viscosity of the ionic liquid is quite high [16]. It is important to state that the activation energy of the nucleation kinetics related to the ion exchange depends on the overpotential used. Even so, the results shown in Table 1, Figure 3a and Figure 4 show that the kinetics of the redox processes of PEDOT films is faster in [EMMIM]Tf_2_N than in acetonitrile [6], which is a relevant characteristic feature for the use of PEDOT thin films to build electrochromic devices or actuators [12,26–27].

### Morphology by AFM

**Figure 5:**
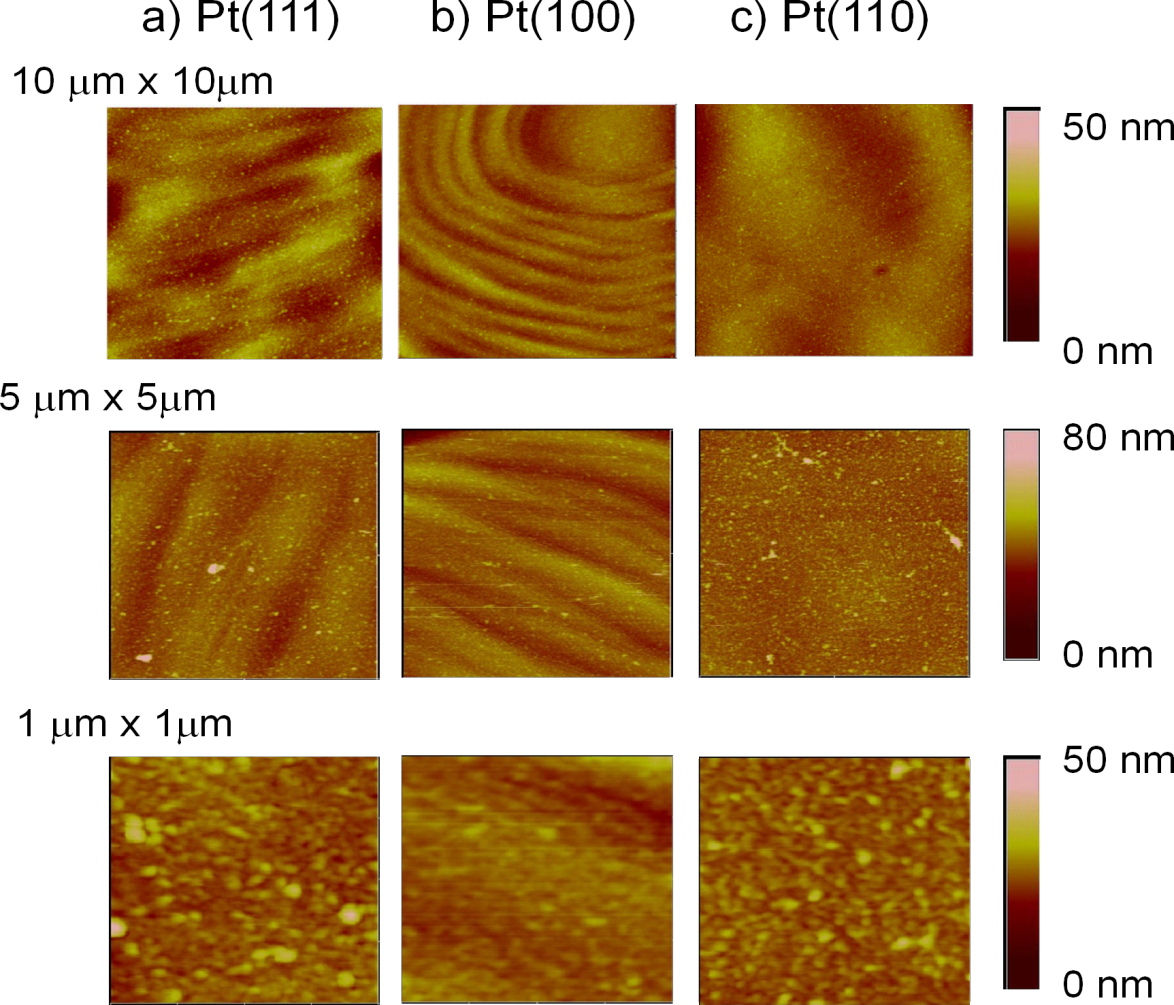
AFM images of PEDOT thin films synthesized on a) Pt(111), b) Pt(100) and c) Pt(110) with a charge density of 1.2 mC cm^−2^. The film thickness is approximately 10 nm.

Figure 5 shows the AFM images of the polymer synthesized with a charge density of 1.2 mC cm^−2^ on the three Pt basal planes. It can be observed a grain shape with a low root mean square roughness (between 3 nm and 4 nm). These results are in agreement with those obtained by MacFarlane et al. [13,28] which also reported very flat polymers in ILs. Opposite to the behavior obtained in aqueous or organic media, the synthesis in ILs generates very flat and homogeneous polymers [29–30]. Specially in the case of Pt(100), the polymer follows the surface and its defects, indicating a two-dimensional growth [31].

### In situ FTIR

When the vibrational behavior of PEDOT films is analyzed by in situ infrared spectroscopy, the observed bands depend on the applied potential. The reference potential in this case was −0.9 V in order to start form the neutral form of PEDOT. Therefore, the positive bands correspond to products present in the oxidized form while the negative bands correspond to species consumption at the working potential.

**Figure 6:**
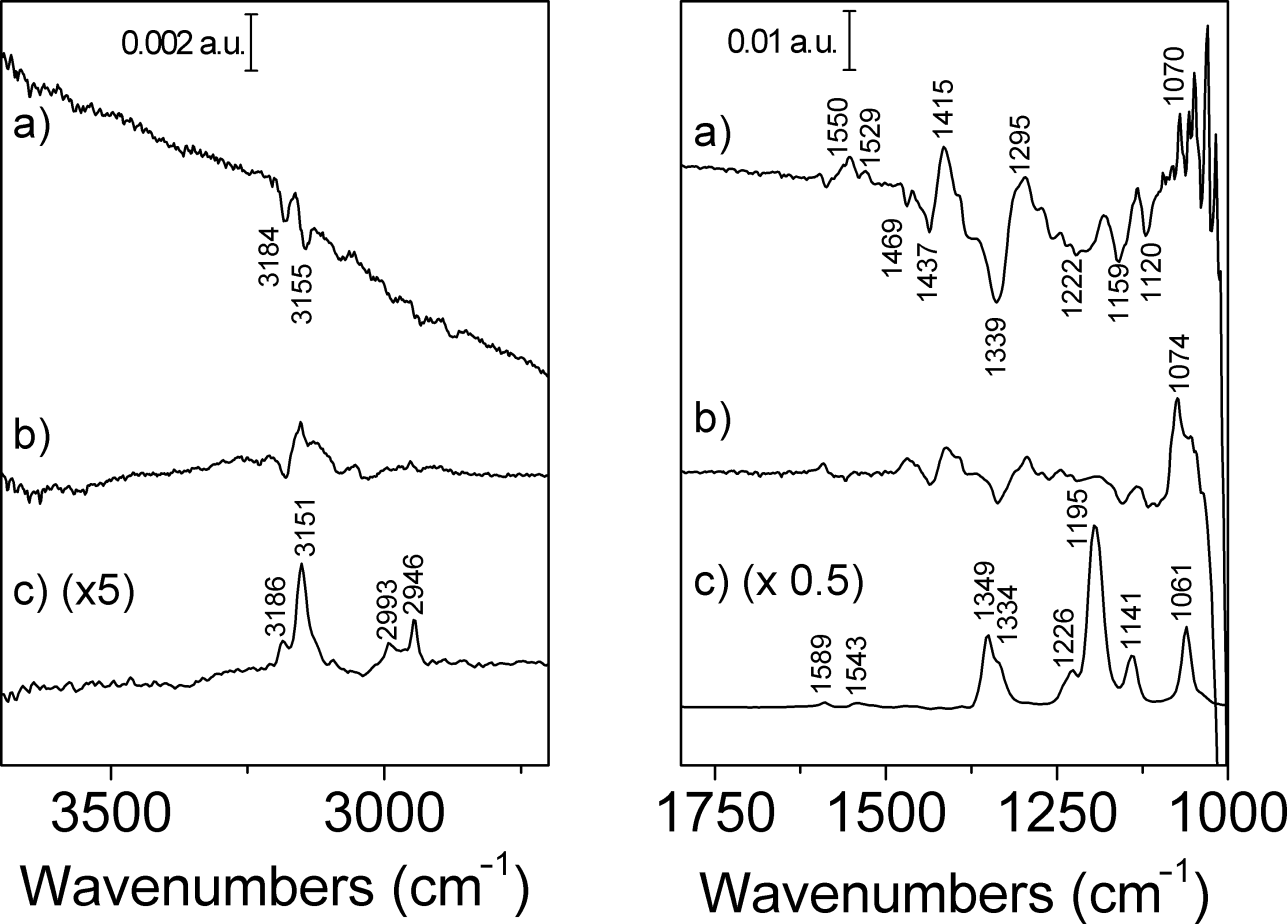
In situ FTIR spectra of a PEDOT thin film synthesized with a charge density of 0.6 mC cm^−2^ on Pt(111). 250 interferograms were recorded per spectrum. The reference potential was −0.90 V. a) p-Polarized spectrum at 0.80 V. b) s-Polarized spectrum at 0.80 V. c) ATR spectrum of 0.1 M [EMMIM]Tf_2_N in acetonitrile, obtained as the average of 100 interferograms.

**Figure 7:**
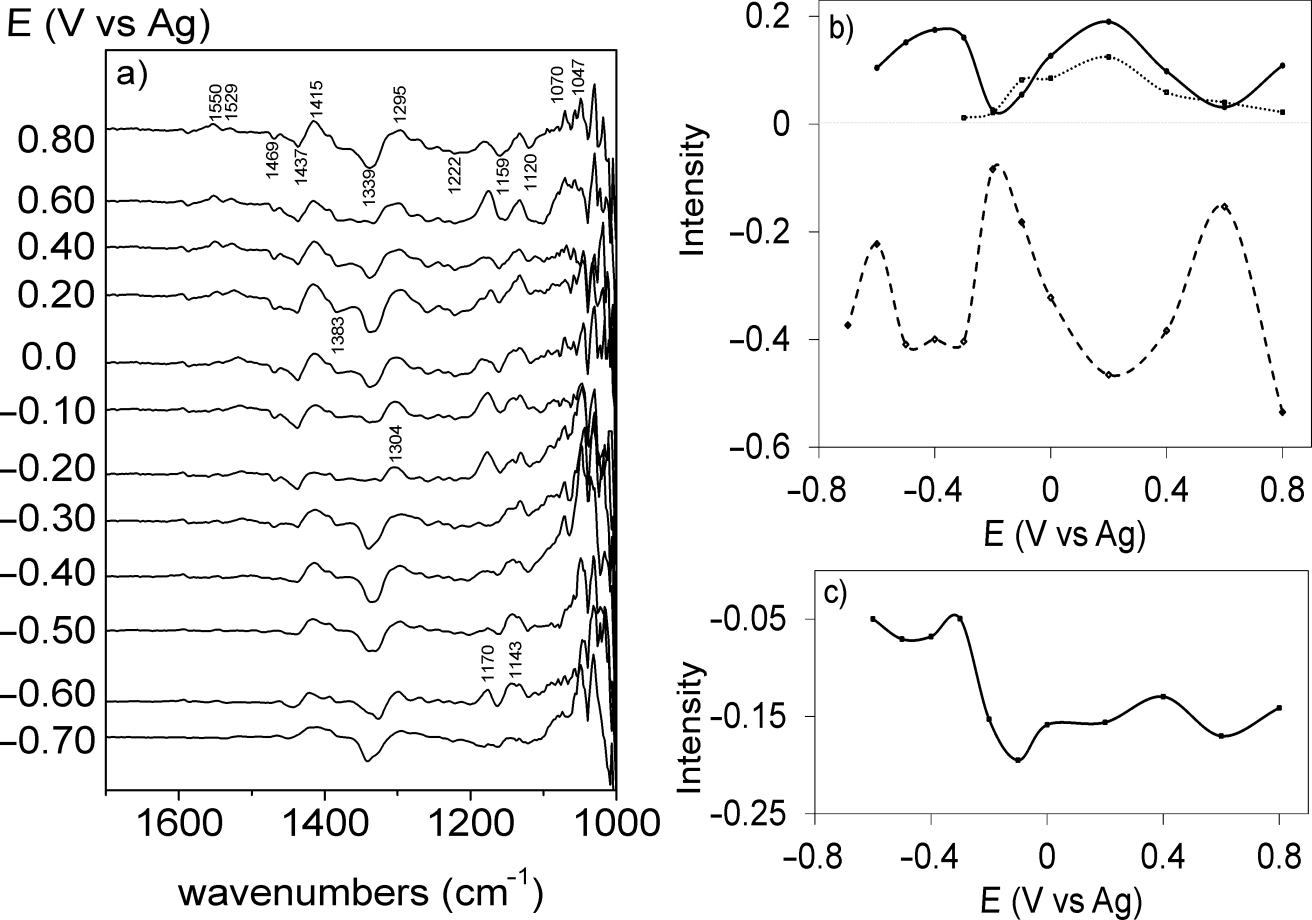
a) In situ FTIR spectra of a PEDOT thin film synthesized with a charge density of 0.6 mC cm^−2^ on Pt(111). 250 interferograms were recorded per spectrum. The reference potential was −0.90 V. Integrated IR intensities of several functional groups vs electrode potential: b) 1415 cm^−1^ (solid line) doping induced band , 1529 cm^−1^ (dotted line) doping induced band; 1339 cm^−1^ (dashed line) v_as_(SO_2_); c) 1437 cm^−1^ (solid line) ν(C=C).

Usually, PEDOT films are studied under attenuated total reflection conditions [17,19,32] because it is difficult to distinguish between the bands coming from solution species and the bands coming from the polymer film. However, we found that it was possible to study the PEDOT behavior under external reflection conditions when very thin layers of polymer are used, as it can be observed in Figure 6 and Figure 7. A comparison between the solution spectrum of the IL and the p- and s-polarized spectra of the polymer is shown in Figure 6. The p-polarized spectrum shows a high absorption between 4000 and 2000 cm^−1^ typical of conducting polymers. This is an electronic absorption related to the formation of charge carriers [3]. However, this feature is not observed with the s-polarized light, which means that the p-polarized spectrum is effectively recording the behavior of the electrode surface. Also, it is important to recognize that there are no water bands, which means that the IL is practically dry.

The bands at 3186 and 3151 cm^−1^ in the p-polarized and ATR spectra, are related to the imidazolium cation. However, the intensity of these bands is very low, hindering their detection. The negative orientation of the bands in the p-spectra means that a considerable amount of cations is being exchanged during the oxidation of the polymer, as it has been reported previously [5].

The zone between 1800 and 1000 cm^−1^ is complex because both polymer and IL bands are detected. The bands at 1334, 1226 cm^−1^, related to the [Tf_2_N] anion, are observed as negative bands both in the p-polarized and the s-polarized spectra which means that the anion is been consumed from the solution. Ispas et al. [33] reported that PEDOT film charge neutralization in ILs proceeds mostly by anion incorporation during the p-doping process, but for [EMIM]Tf_2_N at 85 °C and [EMIM][OTf] at 25 °C the charge regulation occurs mainly via cation exchange. Our results show that, upon oxidation of the PEDOT films in [EMMIM]Tf_2_N both cations and anions are switched but more cations than anions have to be exchanged in order to keep ion neutrality. In other words, it seems that during PEDOT redox cycling some part of the anions remain within the PEDOT film.

The bands related to the oxidation of the polymer and their assignment, based on the literature [6,17,19,32,34], are listed in Table 2. The absorption bands at 1339 cm^−1^ and 1415 cm^−1^ can be assigned to the asymmetric stretching, ν_as_, of the SO_2_ moiety and the stretching of the thiophene ring in the oxidized state, respectively. Figure 7b shows that there are two potential regions where significant changes of the IR peak intensity are observed, between −0.6 V and −0.2 V and between −0.2 V and 0.6 V. These are the same potential zones where the two reduction signals are observed (Figure 3a). The two potential regions mean that there are two different processes, both related to p-doping, which can be related to two different redox or conformational states. It is important to notice that the intensity of these two signals oscillate out of phase in the potential range studied. Also, the band at 1132 cm^−1^ associated with the symmetric stretching of the SO_2_ is positive within the whole potential range showing an adsorption at the surface in which this vibrational mode is active. These features could mean that the consumption of anions (from the IL to inside the polymer) is directly related to the generation of positive charge, which occurs in two regions that correlate well with the peaks A1/C1 and A2/C2 observed in Figure 2.

**Table 2:** Comparison between the absorption bands of ATR spectrum of 0.1 M [EMMIM]Tf_2_N in acetonitrile and external reflection FTIR spectra of PEDOT in [EMMIM]Tf_2_N.

Wavenumber (cm^−1^)		Assignment

[EMIM]Tf_2_N]	PEDOT	

3186	3186 (n)	ν(C-H) ring [34]
3151	3151 (n)	ν(C-H) ring [34]
2993		ν(C-H) alkyl
2946		ν(C-H) alkyl [34]
1589		ν(C=C) ring [35]
1543		ν(CH-N) [35]
	1529 (p)	Doping induced band [17]
	1469 (n)	ν(C=C) [17,19]
	1437 (n)	ν(C=C) [19]
	1415 (p)	Doping induced band
	1381 (n)	ν(C-C) [19]
1349		ν_as_(SO_2_) [34]
1334	1339 (n)	ν_as_(SO_2_) [34]
	1296 (p)	ν_interring_ [17,19]
1226	1222 (n)	ν_s_(CF_3_) [34]
1195		ν_as_(CF_3_) [34]
	1176 (p)	ν(C=C), ν(COROC) [19]
1141	1132 (p)	ν_s_(SO_2_) [34]
	1070 (p)	ν(COROC) [17,19]
1061		ν_as_(SNS) [34]
	1045 (p)	ν(COROC) [17,19]

(n) Negative oriented band; (p) positive oriented band; ν: stretching; s; symmetric; as: asymmetric; COROC: ethylenedioxy group.

Also, the bands associated with the C=C bonds in the thiophene ring (1437 and 1469 cm^−1^) are negative. The trend of the IR intensity at 1437 cm^−1^ (Figure 7c) shows a slight decrease from −0.7 to −0.3V and an abrupt fall at higher potentials. This trend closely resembles the currents observed during the p-doping of PEDOT (Figure 3a), suggesting that the disappearance of the ν(C=C) stretching signal of the thiophene ring upon oxidation can be used to quantify the charge carriers inside the polymer. It seems that with increasing potential, the concentration of charge carriers (polarons) increases between −0.3 V and −0.1 V where a plateau is reached. This IR signal also behaves very similar as the EPR signal intensity upon changing potential reported by Domagala et al. [23].

Finally, it is important to remember that when a weak electric field is applied across a molecule, both the molecular vibrational energy levels and the transition dipoles between the levels change slightly. These changes affect the infrared absorption spectrum of the sample, i.e., the position and the intensity of the bands [36–37]. Taken into account that the IR spectra were recorded at different potentials and that the ion orientation depends on the intensity and direction of the electric field at the interface, some influence of this effect on the spectra shown in Figure 7 is also expected. This effect can explain, in part at least, the complex behavior observed in Figure 7.

## Conclusion

PEDOT films were synthesized on platinum single crystal electrode substrates in [EMMIM]Tf_2_N. Cyclic voltammetry shows that the polymer grows faster in Pt(111) than in Pt(110) or Pt(100). On the other hand, the chronoamperometric experiments show that the activation energy for the oxidation and reduction processes is very low and that the kinetics of the redox processes of PEDOT films is faster in [EMMIM]Tf_2_N than in acetonitrile, which is very useful to build electrochromic devices or actuators.

Pt(111) and Pt(110) show almost the same characteristics in cyclic voltammetry, chronoamperometry and AFM experiments. On the other hand, polymer films grown on Pt(100) present marked differences, which suggests a different nucleation and growth mechanisms in this surface, probably a progressive 2D growth. Finally, the IR and voltammetric experiments show two potential zones where it seems that some changes on the structure and/or on the nature and number of charge carriers take place.

## Experimental

The electrochemical experiments were performed in single compartment glass cells. A platinum wire was used as counter electrode, and a silver wire was used as the pseudoreference electrode. Platinum single crystals were used as working electrodes, which were prepared from small single-crystal beads following the Clavilier’s method [38].

Prior to each experiment the cell was deareated with Ar (≥99.995% Alphagaz). The electrodes were heated in a gas–oxygen flame, cooled down in a reductive atmosphere (H_2_ + Ar) and protected with a droplet of the IL at temperatures low enough to avoid decomposition. Then, the electrode was positioned in contact with the IL using a meniscus configuration.

1-Ethyl-2,3-dimethylimidazolium triflimide ([EMMIM]Tf_2_N), >99% purity, was purchased from Iolitec. It was purified prior to use as it has been previously reported [20].

Polymer films were grown under galvanostatic conditions (unless otherwise stated) by applying a current density of 0.124 mA cm^−2^ during 5, 10, 25, 35 or 50 s in a solution of 0.1 M 3,4-ethylenedioxythiophene (EDOT, Sigma-Aldrich 97%). After the electropolymerization, the PEDOT films were rinsed with ionic liquid free of monomer and then positioned in contact with fresh ionic liquid using a meniscus configuration. 50 scans were performed to ensure a stable voltammetric profile. Characterization was also made in a solution of 0.1 M [EMMIM]Tf_2_N in acetonitrile (Sigma-Aldrich, anhydrous, 99.8%)

A commercially available potentiostat/galvanostat µ Autolab III (Ecochemie) was used for the electrochemical experiments. The morphology of the polymer was studied with a NanoScope III Multimed contact AFM. Scan rate of 2 Hz. A standard cantilever of Si_3_N_4_ with piramidal tips (Digital Instruments) were used.

In situ Fourier Transform Infrared spectroscopy (FTIR) experiments were performed with a Nexus 8700 (Thermo Scientific) spectrometer equipped with an MCT detector. The spectroelectrochemical cell was provided with a prismatic CaF_2_ window beveled at 60°. Spectra shown are composed of 250 interferograms and were collected with a resolution of 4 cm^−1^. Unless otherwise stated, the spectra were collected with p-polarized light. They are presented as absorbance, according to *A* = −log(*R*/*R*_0_), where *R* and *R*_0_ are the reflectance corresponding to the single-beam spectra obtained at the sample and reference potentials, respectively [39]. The contact of the electrodes with the IL was performed at a potential where the polymer was reduced (−0.90 V). This potential was maintained while the electrode was pressed against the CaF_2_ window. After collecting the reference spectrum at this potential, the potential was stepped progressively to higher potentials up to 0.80 V waiting 5 minutes between steps to ensure the stability within the thin layer.

Attenuated total reflection configuration (ATR) was used to obtain the spectra of 0.1 M [EMMIM]Tf_2_N in acetonitrile using a ZnSe hemicylindrical window with an incident angle of 45°. The reference spectrum was obtained in acetonitrile. The spectra are composed by 100 interferograms with a resolution of 8 cm^−1^.
